# Evaluation of multivariate linear regression and artificial neural networks in prediction of water quality parameters

**DOI:** 10.1186/2052-336X-12-40

**Published:** 2014-01-23

**Authors:** Hamid Zare Abyaneh

**Affiliations:** 1 Department of Irrigation and Drainage Engineering, Faculty of Agriculture, Bu-Ali Sina University, Hamedan, Iran

**Keywords:** ANN, MLR, BOD, COD, Wastewater treatment plant

## Abstract

This paper examined the efficiency of multivariate linear regression (MLR) and artificial neural network (ANN) models in prediction of two major water quality parameters in a wastewater treatment plant. Biochemical oxygen demand (BOD) and chemical oxygen demand (COD) as well as indirect indicators of organic matters are representative parameters for sewer water quality. Performance of the ANN models was evaluated using coefficient of correlation (r), root mean square error (RMSE) and bias values. The computed values of BOD and COD by model, ANN method and regression analysis were in close agreement with their respective measured values. Results showed that the ANN performance model was better than the MLR model. Comparative indices of the optimized ANN with input values of temperature (T), pH, total suspended solid (TSS) and total suspended (TS) for prediction of BOD was RMSE = 25.1 mg/L, r = 0.83 and for prediction of COD was RMSE = 49.4 mg/L, r = 0.81. It was found that the ANN model could be employed successfully in estimating the BOD and COD in the inlet of wastewater biochemical treatment plants. Moreover, sensitive examination results showed that pH parameter have more effect on BOD and COD predicting to another parameters. Also, both implemented models have predicted BOD better than COD.

## Introduction

Water is a vital matter for all aspects of human and ecosystem survival and health. Thus, its quality is also important. Evaluation of water quality parameters is necessary to enhance the performance of an assessment operation and develop better management and planning for water resources. The quality of wastewater generated in any process industry is generally indicated by performance indices namely biochemical oxygen demand (BOD) and chemical oxygen demand (COD). The BOD and COD are representative parameters for sewer water quality [1]. The BOD is an approximate measure of the amount of biochemical degradable organic matter present in a water sample and is for domestic wastewater. COD values are always greater than BOD values, but COD measurements can be made in a few hours while BOD measurements take five days [2]. Also, COD is more for industrial wastewater. However, it is very difficult to obtain continuous water quality data due to the scarcity of accessible space within the sewer systems and the necessity of separate laboratory experiments. Currently available method for BOD and COD determination is very tedious and prone to measurement errors. Presence of toxic substances in a sample may also affect microbial activity leading to a reduction in the measured BOD and COD values [3]. Due to the correlations and interactions between water quality parameters, it is interesting to investigate whether a domain-specific mechanism governing observed patterns exists to prove the predictability of these variables [4]. Several water quality models such as traditional mechanistic approaches have been developed in order to manage the best practices for conserving the quality of water [5]. Most of these models need several different input data which are not easily accessible and make it a very expensive and time consuming process [6].

In recent years, Artificial Neural Network (ANN) method has become increasingly popular for prediction and forecasting in a number of disciplines, including water resources and environmental science. The ANN using varied input combinations of quality parameters were trained using various training algorithms. The ANN performance was compared with multivariate linear regression (MLR) approach. The ANNs are able to find and identify complex patterns in datasets which may not be well described by a set of known processes or simple mathematical formula [7].

Dogan et al. studied the abilities of ANN model to improve the accuracy of the biological oxygen demand (BOD) estimation [5]. In this study the potential of an ANN technique in BOD estimation in the Melen river was examined by comparing the results with observed BOD. From the obtained results, an ANN model appears to be a useful tool for prediction of the BOD in Melen river. In study of Guclu and Dursun [8] three independent ANN models trained with back-propagation algorithm were developed to predict effluent COD, suspended solids (SS) and aeration tank mixed liquor suspended solids (MLSS) concentrations of the Ankara central wastewater treatment plant. Elmolla et al. examined the implementation of ANN for the prediction and simulation of COD removal from antibiotic aqueous solution by the Fenton process and is very close to the experimental results with correlation coefficient (R^2^) of 0.997 and mean square error (MSE) of 0.000376 [9]. Estimation of oxygen demand levels using UV–vis spectroscopy and results showed that in most cases the proposed technique of UV-ANN has the best performance. The predicted values of BOD and COD using UV-ANN method were very close to values obtained by using the standard [10]. Dogan et al. developed an ANN model to estimate daily BOD in the inlet of wastewater biochemical treatment plants [5]. In this study, The ANN technique with COD, water discharge, suspended solid, total nitrogen, and total phosphorus presented MSE of 708.01, average absolute relative errors of 10.03%, and a coefficient of determination of 0.919. Onkal-Engin et al. used ANN for determination of the relationship between sewage odor and BOD [11]. Their results showed that ANNs can be used to classify the sewage samples collected from different locations of a wastewater treatment plant. Rene and Saidutta applied ANNs to predict the concentrations of BOD and COD by using some easily measurable water quality indices [12]. Their results showed that the ANN ability in prediction of BOD was better than COD. Oliveira-Esquerre et al. developed multilayer perceptron (MLP) and functional-link neural networks (FLN) to predict inlet and outlet BOD of an aerated lagoon operated by International Paper of Brazil [13]. They reported MLP networks are the best choice for the prediction of BOD. Akratos et al. applied ANN model and design equations for BOD and COD removal prediction in horizontal subsurface flow constructed wetlands [14]. Results of the ANNs and the model design equation were close to experimental data from the literature. Results showed that a rather satisfactory correlation was obtained using ANN method. COD removal was found to be strongly correlated to BOD removal. An equation for COD removal prediction was also produced.

Due to numerous problems in the registration and measurement of water quality such as BOD and COD, the main aim of the present study was: 1) to find the optimized topology of the ANN and new regression models for prediction of complex water quality data; 2) to select the best method in prediction of the water quality data, and 3) to evaluates the results of the multilayer perceptron type ANN in prediction of BOD and COD removals and selecting the optimized topology.

## Material and methods

### Study area

**Figure 1:**
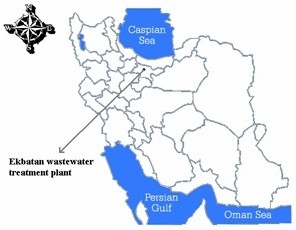
Location of the study area.

The data set used in this study was gathered through continuous monitoring of samples from Ekbatan wastewater treatment plant, Tehran, Iran (51° 15′ 45′ E, 35° 42′ 10′ N). Figure 1 shows the location of the study area. The water quality parameters were measured in water quality laboratory of refinery from 1998 to 2002. Based on measured values of different variables and their correlative analysis, factors (variables) including pH, total suspended solid (TSS), total suspended (TS) and water temperature (T) were identified which affect the water quality (BOD and COD) and finally selected for the model development. BOD is determined by incubating a sealed sample of water for five days and measuring the loss of oxygen from the beginning to the end of the test. Samples often must be diluted prior to incubation or the bacteria will deplete all of the oxygen in the bottle before the test is complete. The COD value was determined by the procedures described in the Standard Methods of APHA [15]. The monthly measuring of six water quality parameters was conducted during 7 years (2003–2009).

Boundary of the water quality parameter values in the model domain is given in Table 1. In this table, X_mean_, X_max_, X_min_, SD_x_ and CV indicate the mean, maximum, minimum, standard deviation and deviation coefficient of the data set, respectively.

**Table 1:** Water quality properties in the ANN and MLR model domain measured during period 2003–2009 in Ekbatan wastewater treatment plant

**CV**	**SD**_**x**_	**X**_**min**_	**X**_**max**_	**X**_**mean**_	**Unit**	**Data set**
0.04	0.3	7.2	8.7	7.9	— —	pH
0.10	2.4	18.5	27.3	23.7	^0^C	T
0.14	93.2	400	944	646.4	mg/L	TS
0.43	106.5	75	568	245.9	mg/L	TSS
0.28	45	50	249.0	159.1	mg/L	BOD
0.35	89.8	80	502	257.6	mg/L	COD

As can be seen in Table 1, the variation coefficient (CV) of BOD and COD are 0.28 and 0.35. The CV is a statistical dispersion index of data set, which is defined as mean normalized standard deviation of data set. The CV formula is as follows:

(1) $$\mathrm{CV}=\frac{SD_{x}}{X_{\mathrm{mean}}}$$

Variation coefficient values of BOD and COD in this study are lower than other studies. In the study of Singh et al. concentration of both water quality parameters showed large variations between the samples, with a high variation coefficient (0.48 for COD and 0.83 for BOD) [3]. Such differences may be attributed to the large geographical variations in climate, number of samples and water quality in the study region. The CV of pH compared with other parameters is very low (0.01). Different variation coefficient returns to the nature of parameters. Similar to Singh et al. the computed variables of anthropogenic origin showed larger variations as compared to the natural origin variables. This may be attributed to the fact that the geogenic processes are almost in equilibrium state, whereas, the anthropogenic processes are time dependent in nature [3].

### Artificial neural network (ANN)

**Figure 2:**
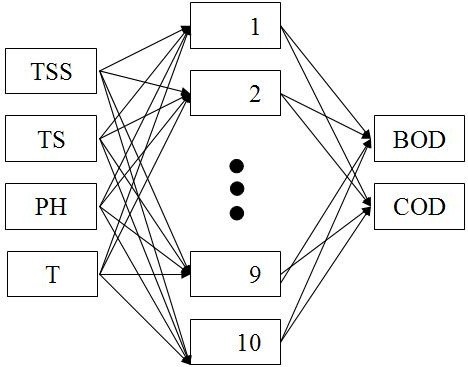
Architecture of the neural network model used in this study.

The ANN models are increasingly being used for forecasting or simulating water resources variables because they are often capable to model complex systems with unknown or difficult behavioral rules or underlying physical processes. The ANN is a non-linear modeling tool capable of handling a large number of inputs (independent variables) to determine one or more outputs (dependent variables) [10]. There are many types of neural networks for various applications available in researches. The multilayer perceptron (MLP) is a widely used ANN configuration and has been frequently applied in the field of hydrological modeling [16]. This study evaluates the utility of MLP neural networks for estimating BOD and COD. The MLP is the simplest and therefore most commonly used neural network architectures [17]. Figure 2 provides an overview of the structure of this network. The MLP consists of three layers of neurons: (1) an input layer; (2) an output layer, and (3) intermediate (hidden) layer or layers. Each neuron has a number of inputs (from outside the network or the previous layer) and a number of outputs (leading to the subsequent layer or out of the network). A neuron computes its output response based on the weighted sum of all its inputs according to an activation function [18].

A simple MLP was used in this study. It is a network with four input variables, a hidden layer with four to a maximum of ten processing neurons and two output variables (BOD and COD). For a simple regression analysis the units in the input layer introduce normalized or filtered values of each input variable into the network, then these values are transferred to all units of the hidden layer multiplied by a “weight” factor that is, in general, different for every connection, and its magnitude characterizes the importance of some connections (Figure 2).

In the present study, two training algorithms (i.e. Levenberg–Marquardt and Momentum) were applied to train the network. Two different transfer functions (i.e. Sigmoid and Tanh) were also used to obtain the best results with respect to non-linearity of this phenomenon. Finally, the best learning algorithm, activation function and architecture of the network (the number of neurons in hidden layers) were determined by trial and error.

For ANN modeling, the experimental data set were divided into a training set (80% of the data) and validation set (20% of the data set). The training set is used to fit ANN model weights (for a number of different network configurations and training cycles). The validation set is used to evaluate the optimized model against unknown data set.

Training and testing of the network were accomplished on NeuroSolutions version 5. In NeuroSolutions, the criteria used to evaluate the fitness of each potential solution are the lowest cost achieved during the training run [19]. To avoid over training, early stopping technique was used in training [20]. This method is done automatically in NeuroSolution software. So that, as soon as over training of ANN, ANN training stops.

### Multivariate linear regression (MLR)

Statistical methods, such as regression models, are the best tools for investigating any relationship between dependent and independent variables of small sample size [21]. The MLR is a method used to model the linear relationship between a dependent variable and one or more independent variables. MLR is based on least squares. In the best model, sum of square error between observed and predicted parameters should be minimum value.

BOD and COD estimation also can be performing using linear models which explain linear relationship between parameters. Furthermore, the same input variables for MLR models are considered for linear models (Eq. 2):

(2) Y=aTSS+bTS+cPH+dT+e

Where, Y: BOD or COD values, a, b, c, d and e: constant coefficients of linear regression model, TSS, T, TS and pH are input parameters.

### Evaluation criteria for ANN and MLR prediction

Two statistical criteria were applied to evaluate the performance of ANN and MLR methods. These criteria were coefficient of correlation (r) and root mean square error (RMSE).

Correlation of coefficient (r) is a common criterion for goodness of fit for regression models [21]. Two additional criteria were used to compare the output values of the models. These criteria are as follow:

(3) $$\mathrm{RMSE}=\sqrt{\frac{1}{n}\sum_{i=1}^{n}{\left(X_{i}-Y_{i}\right)}^{2}}$$

(4) $$r=\frac{\sum \left(X_{i}-\bar{X}\right)\left(Y_{i}-\bar{Y}\right)}{\sqrt{\sum {\left(X_{i}-\bar{X}\right)}^{2}{\left(Y_{i}-\bar{Y}\right)}^{2}}}$$

where *Xi* and *Yi* are the *i*th observed and estimated values, respectively; $\bar{X}$ and $\bar{Y}$ are the average of *X*_*i*_ and *Y*_*i*_*,* and n is the total numbers of data.

## Resuls and discussion

**Figure 3:**
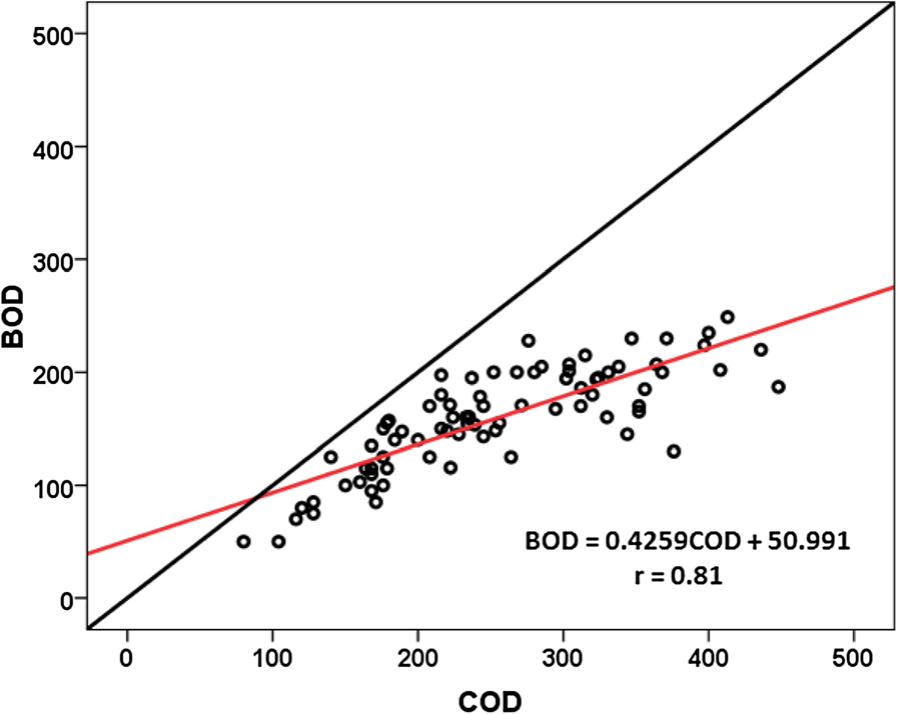
Correlation between measured BOD and COD.

Measured BOD removals are depicted against the corresponding measured COD removals in Figure 3. A simple linear model (y = ax + b) is fitted to data. Akratos et al. [14] obtained a similar model between COD and BOD as follows:

(5) BOD=0.4259COD+50.991r=0.81

This result showed that the BOD removal could be predicted by applying the correlation between BOD and COD removal to the predictions of COD removal. Therefore COD was found to be strongly correlated to BOD. Study of Akratos et al. proved that a strong correlation exists between BOD and COD values [14]. This finding confirms the results of this study.

**Figure 4:**
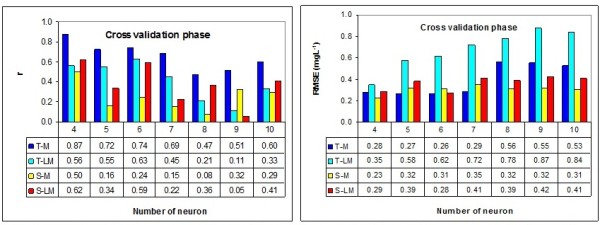
Comparison of convergence speeds for the momentum (M) and Levenberg-Marqurdt (LM) algorithms with sigmoid function (s) and tanh function (T), as measured by number of neurons during validation phase; by Correlation Coefficient (r), root mean squared error (RMSE).

The statistical criteria of RMSE and r for each ANN structure in validation phase are given in Figure 4. Figure 4 shows RMSE of the structure of sigmoid transfer function and four neurons with momentum learning algorithm is the lowest value. Moreover correlation coefficient value of this structure is low. Comparing RMSE and r values for all neural network structures, an optimized structure with neurons in middle layer with momentum algorithm and tanh function was selected. The momentum algorithm adds inertia to the training procedure, and helps avoid oscillatory entrapment in local minima [22]. This structure has the highest correlation value (r = 0.74) and the least error (RMSE = 0.26 mg/L for normal data). Thus it can be stated with 74% confidence, ANN results is acceptable. The remaining 26% can be caused by environmental and climate factors affects on the value of BOD and COD. Also, in the choice structure, the both BOD and COD parameters can be estimated simultaneously. This method reduced the time cost and is recommended.

**Figure 5:**
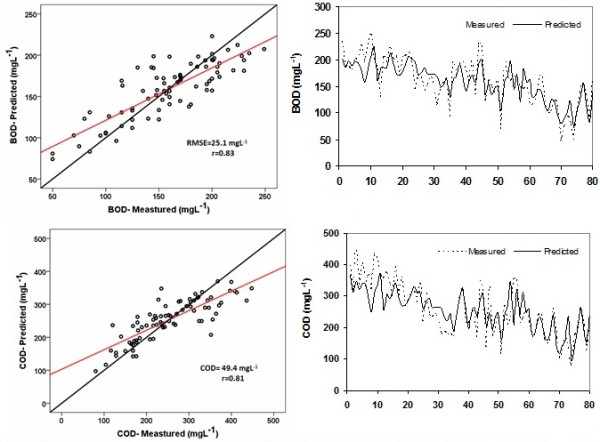
ANN predictions of concentrations of BOD and COD for all of data set.

With regard to the optimized ANN structure, estimated and observed values of BOD and COD as well as time series and linear model fitted to the data were presented in Figure 5.

A comparison of the observed and estimated BOD and COD concentrations as hydrograph and scatter plot form is shown in Figure 5. It can be seen from the hydrographs that the ANN BOD estimates closely follow the observed values. This is also confirmed by the scatter plots (Figure 5). It can be clearly seen from the scatter plots that the BOD has a higher r value (0.83) than COD. This may be due to the fact that the optimized ANN can estimate the BOD with higher precision than COD, due to the higher variation coefficient for COD in relation to the BOD (Table 1).

The low variation coefficient of a parameter is indicative of the high uniformity, which can enhance the accuracy of prediction parameter. As can be seen in Table 1, the variation coefficient of BOD parameter and COD are 0.28 and 0.35, respectively that is high probability indicative of BOD estimating to COD. Also, there exists a better relationship between the BOD and qualitative parameters. Moreover, the accuracy of the COD prediction is acceptable. Figure 5 proved that artificial neural network is suitable for estimating BOD and COD. Furthermore there is a good correlation between estimated and measured values. The difference between the measured and calculated values in some parts is due to the influence of other factors (except 4 input parameters) on the output parameters. As is known, affecting factors on BOD and COD values are not only 4 input parameters used in this study, but other quality factors and climate could be involved that in this research isn’t used. Because, the purpose of investigation was to estimate BOD and COD with using simple minimum parameters. Therefore, although the use of more parameters, it can reduce the difference between the estimated values and observations, but the cost must be justified. While the results of this study in compared to other studies is better. In study of Guclu and Dursun, correlation coefficient was calculated as 0.85 for COD modeling [8]. They used 8 input parameters include flow rate, return activated sludge and waste activated sludge, DO, COD, SS, total kjeldahl nitrogen (TKN) and COD load in modeling process but in the present study four parameters were used. These results indicate that the ANN model has the best performance. Pai et al. found the prediction accuracy at 48.22% for COD [23]. Other studies applied the ANN modeling method to estimate the full-scale wastewater treatment plant [24]. In this study, correlation coefficient (R-square) values were ranging from 0.63 to 0.81 for BOD. Another study showed that the coefficient of correlation values of selected ANN (11 nodes in input layer inclute: water pH, total alkalinity, total hardness, total solids, COD, ammonical nitrogen, nitrate nitrogen, Cl, PO4, K, Na) for forecasting of BOD was 0.87 [3]. Prediction of dissolved oxygen using ANN method indicated that ANN structure with 10 input parameters (pH value, BOD, COD, SS, TKN, ammonia nitrogen, nitrite nitrogen, nitrate nitrogen, total phosphorous and total coliform provides accurate results with r = 0.84 [7]. In the present study, only four input parameters which are easy to measure were successfully used for BOD and COD estimation.

**Figure 6:**
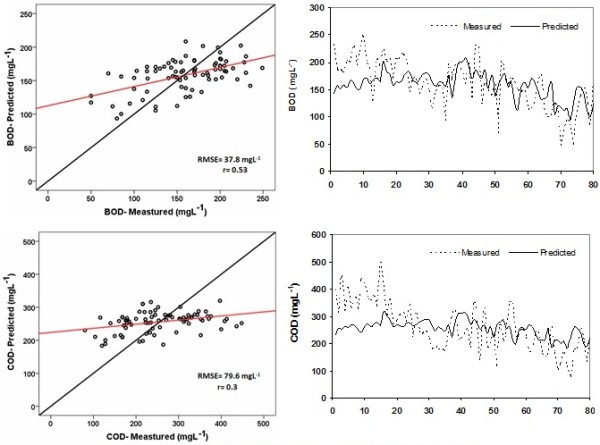
MLR predictions of BOD and COD concentrations for the total data.

Comparison of the observed and estimated BOD and COD concentrations by MLR in hydrograph and scattered forms is shown in Figure 6.

From the root mean square errors and correlation coefficients presented in Figure 6 the observed BOD values are more strongly correlated to the predictions than the observed COD values. Values of r and RMSE for BOD were 0.53 and 37.8 mg/L respectively. Also, for COD r = 0.3 and RMSE = 79.6 mg/L. The optimal results for ANN and MLR models are r = 0.83 versus r = 0.53 for BOD and r = 0.3 versus r = 0.81 for COD, respectively. Comparison between MLR and ANN results in forecasting of the quality parameters showed that the ANN model has less error value than MLR (for example, RMSE = 37.8 mg/L and RMSE = 25.1 mg/L). So the ANN model has better performance than the MLR model. Although study results of May and Sivakumar indicated that multiple linear regression models were more applicable for predicting urban storm water quality than ANN models [25]. Moreover, the ease of regression models run is no secret for anyone. However in most studies, ANN results were better than another models in prediction of water quality parameters [3,5,8,10,14].

**Figure 7:**
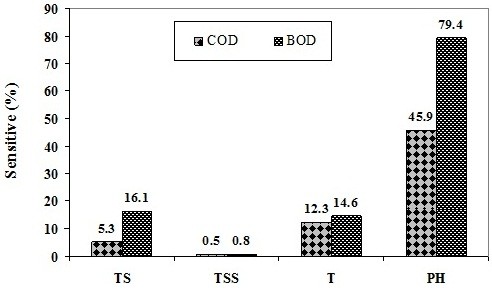
Sensitivity of ANN input parameters in BOD and COD forecasting.

In order to show which one of input parameters is more sensitive, sensitivity of ANN input parameters in BOD and COD forecasting is presented in Figure 7.

Figure 7 shows that ANN results in predicted of BOD and COD have more sensitive to pH parameter. The value of sensitive to pH for BOD and COD are 79.4% and 45.9%, respectively. These values indicates BOD sensitive to pH is more than COD. So, in order to access to better ANN results should measure pH parameter with high careful in compared to another parameters. Importance of pH parameter on BOD and COD, have been reported in study of Verma and Singh [26]. In contrast, the both BOD and COD have lowest sensitive to TSS parameter.

## Conclusions

In the present study, the efficiency of MLR and ANN models were investigated in prediction of two major water quality parameters, BOD and COD, in Ekbatan wastewater treatment plant, Tehran, Iran. Performance of the models was evaluated using coefficient of correlation (r) and root mean square error statistics (RMSE). The results indicated that the ANN model with minimum input parameters, temperature (T), pH, total suspended solid and total suspended could be successfully used for predicting BOD and COD concentrations. It was found in the present study that ANN model trained with momentum algorithm is an effective adsorbent for the prediction of COD and BOD concentrations. The choice structure had the highest correlation value (r = 0.74) and the least error (RMSE = 0.26 mg/L for normal data). Comparison of the ANN and MLR models showed that the ANN model performed much better than the MLR (for example, RMSE = 37.8 mg/L in contrast RMSE = 25.1 mg/L). In both models, predictions of the BOD concentrations with ANN and MLR models were found to be better than COD. Comparing these results with other studies showed that although the minimum easy parameters used in this study, but expected results were better than previous studies. This result suggests that the use of more input parameters will not necessarily lead to improvements of predicted results, but type of input parameters is more important than it’s number.

## Competing interests

The author declares that he has no competing interests.
